# CACNA1A-Linked Hemiplegic Migraine in GLUT 1 Deficiency Syndrome: A Case Report

**DOI:** 10.3389/fneur.2021.679354

**Published:** 2021-05-31

**Authors:** Chiara Scoppola, Giorgio Magli, Marta Conti, Maria Fadda, Giovanni M. Luzzu, Delia M. Simula, Alessandra Carta, Stefano Sotgiu, Susanna Casellato

**Affiliations:** ^1^Center for Diagnosis and Care of Pediatric Epilepsy, University Hospital of Sassari, Sassari, Italy; ^2^Department of Medical, Surgical and Experimental Sciences, Section of Child Neuropsychiatry, University of Sassari, Sassari, Italy

**Keywords:** GLUT1 deficiency syndrome, hemiplegic migraine, *CACNA1A*, epilepsy, headache, SLC2A1

## Abstract

**Background:** Glucose-transporter-1 deficiency syndrome (GLUT1-DS), due to *SLC2A1* gene mutation, is characterized by early-onset seizures, which are often drug-resistant, developmental delay, and hypotonia. Hemiplegic migraine (HM) is a rare form of migraine, defined by headache associated with transient hemiplegia, and can be caused by mutations in either *CACNA1A, ATP1A2*, or *SCN1A*. Paroxysmal movements, other transient neurological disorders, or hemiplegic events can occur in GLUT1-DS patients with a mild phenotype.

**Case:** We report on a girl with GLUT1-DS, due to *SLC2A1* mutation, with a mild phenotype. In early childhood, she developed epilepsy and mild cognitive impairment, balance disorders, and clumsiness. At the age of 9, the patient reported a first hemiplegic episode, which regressed spontaneously. Over the next 3 years, two similar episodes occurred, accompanied by headache. Therefore, in the hypothesis of HM, genetic testing was performed and *CACNA1A* mutation was identified. The treatment with Lamotrigine avoided the recurrence of HM episodes.

**Discussion:** To our knowledge, among the several cases of GLUT1-DS with HM symptoms described in the literature, genetic testing was only performed in two of them, which eventually proved to be negative. In all other cases, no other genes except for *SLC2A1* were examined. Consequently, our patient would be the first description of GLUT1-DS with HM due to *CACNA1A* mutation. We would emphasize the importance of performing specific genetic testing in patients with GLUT1-DS with symptoms evocative of HM, which may allow clinicians to use specific pharmacotherapy.

## Introduction

Glucose-transporter-1 deficiency syndrome (GLUT1-DS), due to *SLC2A1* gene mutation, is characterized by seizures, developmental delay, and hypotonia, although the clinical picture can vary between mild to severe phenotypes (1, 2).

Additional symptoms in GLUT1-DS might consist of paroxysmal movements or other transient neurological disorders. Notably, among them, patients with a GLUT1-DS mild phenotype can also experience transient hemiplegic events (3).

Hemiplegic migraine (HM) is a rare form of migraine, which can be caused by mutations in either *CACNA1A, ATP1A2*, or *SCN1A*. HM attacks are characterized by headache associated with transient neurological deficits such as hemiparesis, impairment in speech, and sensory and visual disturbances (4–6).

We report on a girl presenting with *SLC2A1* and *CACNA1A* mutation.

## Case Description

**Figure 1:**
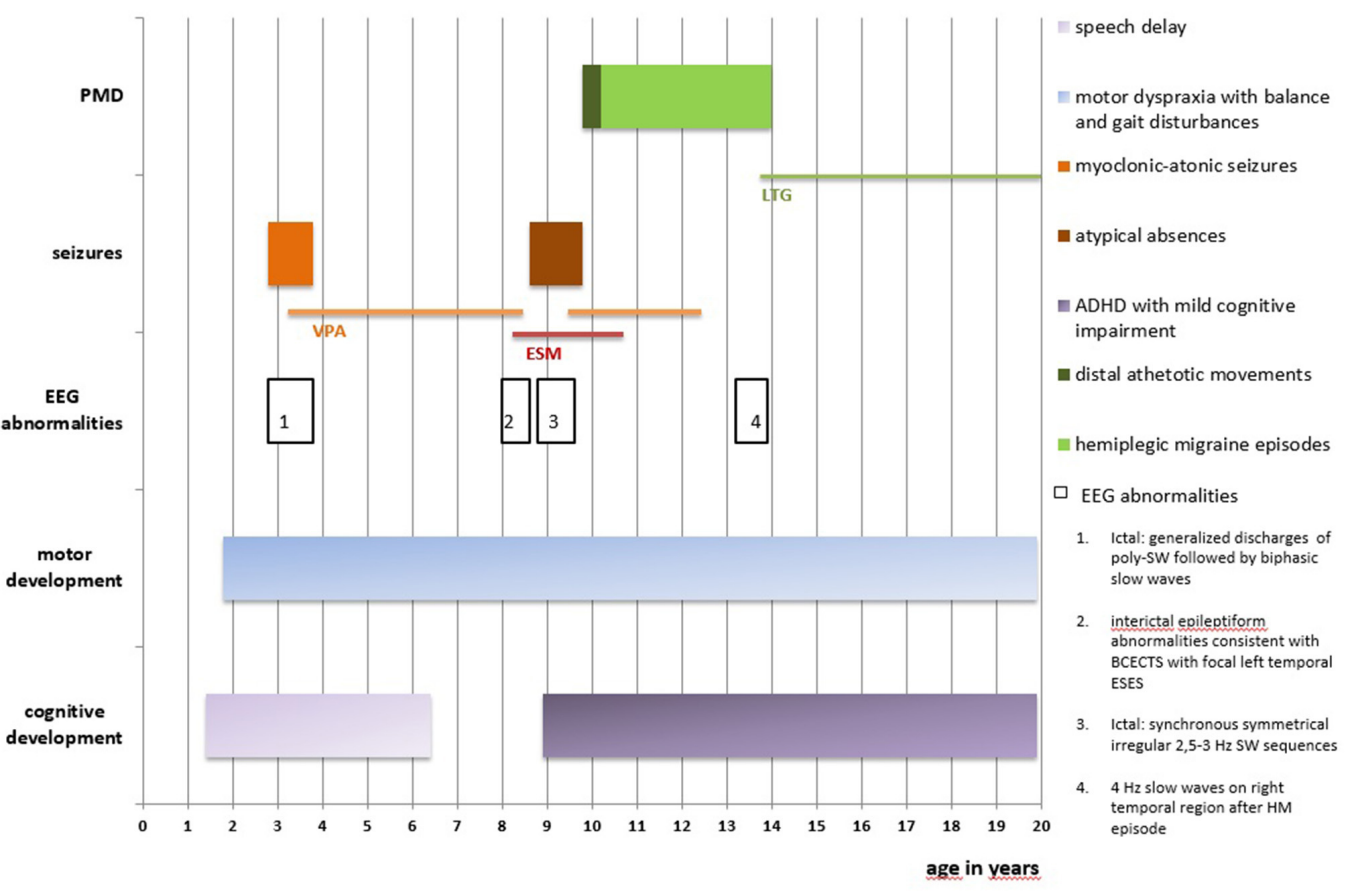
Clinical history is summarized in this figure. PMD, paroxysmal movement disorders; EEG, electroencephalography; BCECTS, Benign Childhood Epilepsy with Centro-Temporal Spikes; ESES, Electrical Status-Epilepticus during slow-waves Sleep; SW, spike-and-wave; poly-SW, polyspike-and-wave; HM, hemiplegic migraine; ADHFD, Attention Deficit and Hyperactivity Disorder; VPA, Sodium Valproate; ESM, Ethosuximide; LTG, Lamotrigine.

A 19-year-old girl who had experienced the onset of frequent sudden falls from the age of 34 months was admitted to the Unit of Child Neuropsychiatry, University Hospital of Sassari (clinical history summarized in Figure 1). On neurological examination, psychomotor development featured a slight delay, together with motor dyspraxia and a wide-based gait. A follow-up evaluation performed at 3 years and 5 months pointed out mild speech delay and dyslalia.

At age nine, Wechsler Intelligence Scale for Children-III (WISC-III) showed total I.Q. of 55 (Verbal I.Q. 59, Performance I.Q. 60) and a diagnosis of Attention Deficit and Hyperactivity Disorder, combined type (ADHD-C), with mild cognitive impairment, was formulated.

Seizures started at the age of 2 years and 10 months, characterized by sudden falls to the ground or sudden head drops, associated with loss of awareness, often anticipated by mild distal myoclonias involving both upper and lower limbs. These episodes were classified as myoclonic-atonic seizures. At seizure onset, electroencephalography (EEG) showed normal awake and sleep background activity; ictal EEG showed diffuse discharges of polyspike-and-wave 300–400 microV, followed by biphasic slow waves, synchronous with clinical myoclonic-atonic episodes. Monotherapy with Sodium Valproate (VPA) was effective on seizures' control and normalization of the EEG records.

Brain and spinal cord MRI, metabolic screening, electroneurography, electrocardiogram, and echocardiography were all reported to be normal.

At the age of eight, follow-up EEGs showed interictal epileptiform abnormalities consistent with Benign Childhood Epilepsy with Centro-Temporal Spikes (BCECTS) with focal left temporal Electrical Status-Epilepticus during slow-waves Sleep (ESES). Therefore, Ethosuximide (ESM) was prescribed, while VPA was gradually discontinued.

**Figure 2:**
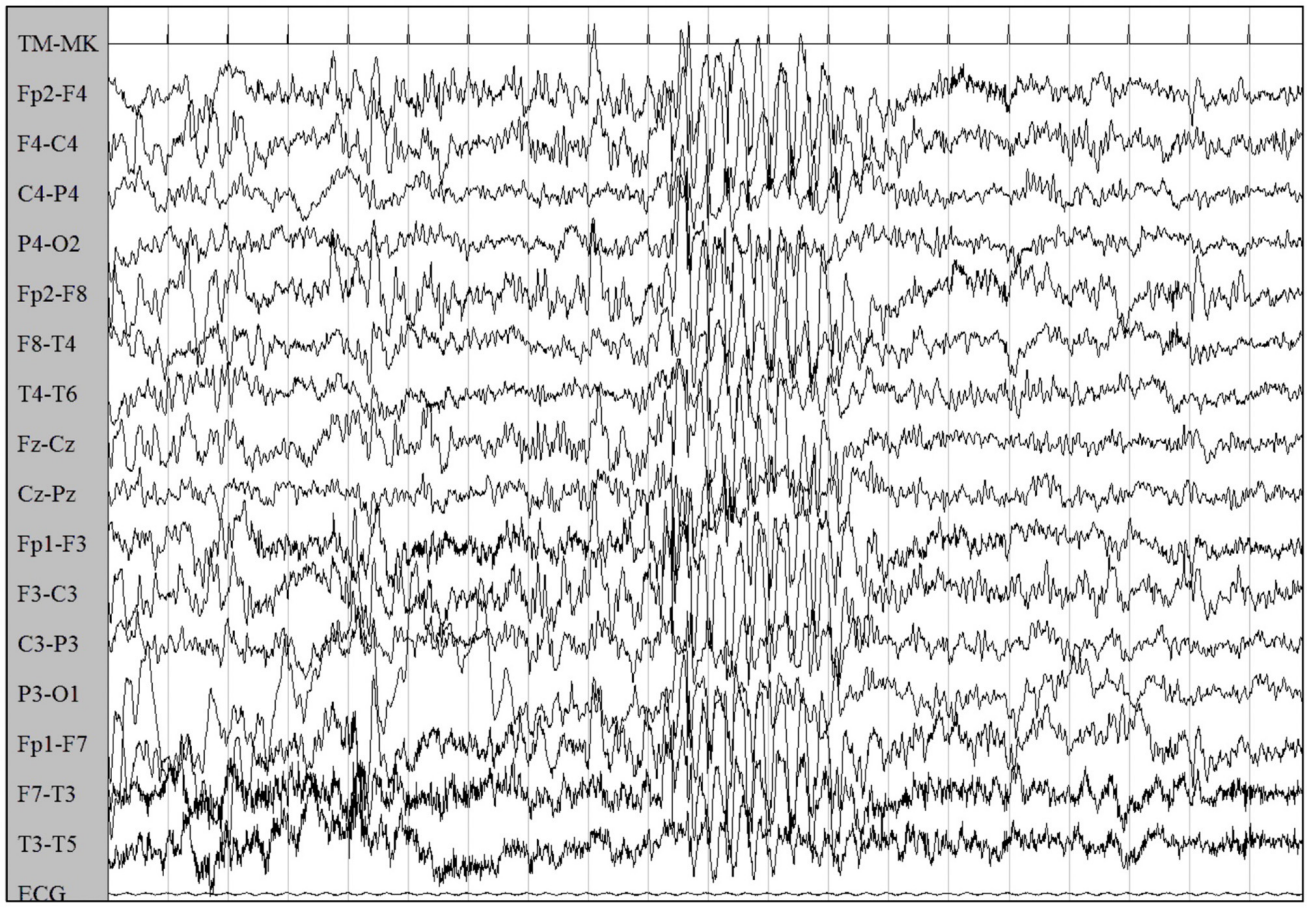
EEG showed synchronous symmetrical irregular 2.5–3 Hz spike-and-wave sequences, facilitated by hyperventilation, consistent with atypical absence seizure.

Six months later, concurrently with the complete withdrawal of VPA, episodes of staring and loss of awareness lasting 3 s were reported. EEG showed synchronous symmetrical irregular 2.5–3 Hz spike-and-wave sequences lasting 2 s, facilitated by hyperventilation, consistent with atypical absence seizures (Figure 2).

Treatment with VPA was then restored, while ESM was gradually discontinued, given the age-related disappearance of ESES and abnormalities consistent with BCECTS and its ineffectiveness on absences seizures. VPA treatment was successfully maintained for the following 3 years and discontinued after being clinically and EEG seizure-free for 2 years.

At age 9, the patient experienced episodes of right hemiparesthesia and dysarthria lasting up to 25 min, with spontaneous regression, together with episodes of sudden distal athetotic movements of hands and feet, without EEG abnormalities (Supplementary Video 1). Neurologic examination confirmed the persistence of the balance and gait disturbances (i.e., wide-based gait).

The clinical complexity led to the hypothesis of GLUT1-DS. Thus, the genetic investigation was performed and confirmed the presence of a heterozygous c.667C>T *de novo* mutation of exon 5 in the *SLC2A1* gene, given that the same mutation was not detected in the parents' blood sample.

A ketogenic diet was initiated and discontinued, after two attempts, owing to poor compliance.

Further non-epileptic paroxysmal episodes presented at age 13, the first of which was characterized by right hemiparesthesia and dysarthria, combined with cephalalgia and emesis, followed by hemiparesis lasting 90 min. On another occasion, the girl experienced the same symptoms on the left hemisoma, without dysarthria. The EEG performed 2 days after this last episode showed 4 Hz slow waves, 40–60 microV, on the right temporal region, which disappeared on subsequent EEGs.

Due to the suspicion of HM, specific genetic testing was carried out, leading to the identification of a heterozygous c.3043G>A, p.(Glu1015Lys) mutation in the *CACNA1A* gene. We have not performed genetic testing for this mutation on the parents as the anamnesis was negative for episodes of migraine and HM.

Treatment with Lamotrigine was initiated with complete remission of HM episodes. Follow-up EEGs have been normal since then.

## Discussion

Glucose-transporter type 1 (GLUT1), encoded by *SLC2A1* gene on chromosome 1, is a transmembrane protein, which allows glucose to pass across the blood-brain barrier (BBB). *SLC2A1* mutations cause GLUT1-DS, resulting in impaired glucose transport across the BBB and consequent hypoglycorrhachia (1).

GLUT1-DS occurs with variable clinical phenotypes, which are usually classified into two groups: “classical” or severe and “non-classical” or mild phenotype. The severe form is characterized by microcephaly, infantile-onset epilepsy, which is often drug-resistant, intellectual disability, and disabling movement disorders; conversely, the mild phenotype involves paroxysmal movement disorders, childhood epilepsy with atypical absences, and/or myoclonic-atonic epilepsy (1–3).

Hence, GLUT1-DS is categorized as developmental and epileptic encephalopathy (DEE) (7).

Symptom severity depends on glucose intake, with symptoms worsening during fasting and improving with meals. Moreover, clinical signs can often vary in the single patient's history, which makes the diagnosis even more challenging. Generally, epileptic seizures precede the onset of movement disorders and, in some cases, a progressive remission of epilepsy is described; it is likely that modifications of glucose metabolism in the cerebral cortex, related to growth, could explain symptoms' heterogeneity and variability over time (2).

Three different types of paroxysmal movement disorders (PMD) are described in GLUT1-DS, namely exercise-induced paroxysmal dyskinesia, which is the most frequent, paroxysmal kinesigenic dyskinesia, and paroxysmal non-kinesigenic dyskinesia (PNKD). In 10% of GLUT1-DS identified as mild phenotype, the main clinical manifestation is PMD (3).

The ketogenic diet represents a specific therapy for GLUT1-DS, as it improves epilepsy management, PMD, and cognitive development, especially if it is started in the early stage of the disease (1).

Hemiplegic Migraine (HM) is a rare type of migraine, featuring transient neurological deficits such as hemiparesis, speech impairment, and sensory and visual disturbances, often accompanied by headache (4–6). Patients are mostly females, with the onset occurring mostly in their first or second decade and an age-related decrease of attack frequency (4, 5).

In the International Classification of Headache Disorders-3, two different HM subtypes are included: familial and sporadic hemiplegic migraine (6).

Mutations in the ion transportation genes *CACNA1A, ATP1A2*, and *SCN1A* are well-known to cause HM; despite this, mutations in these genes are found in <20% of HM cases (4, 8). Other genes with roles in synaptic function and neurotransmission have also been implicated in the pathogenesis of HM and HM-like disorders such as *PRRT2* mutations (4, 8, 9). The management of HM is not specific; among the most commonly used prophylactic and effective treatments, Verapamil, Lamotrigine, Flunarizine, Naloxone, and Acetazolamide are described (4, 5).

Transient hemiplegic episodes, resembling HM, can be experienced in patients with a GLUT1-DS, particularly in those with mild phenotype, although only nine cases of GLUT1-DS with HM attacks are described in the literature (3, 9–14). To our knowledge genetic testing for HM was only performed in two of them, which eventually resulted to be negative (3, 12). In all other cases, only the *SLC2A1* gene was examined (9–11, 13, 14).

Moreover, in a large population of HM patients with no mutation on the main genes (*CACNA1A, ATP1A2*, or *SCN1A*), 1.3% of cases presented with a mutation on *SLC2A1* and having HM episodes as the main clinical feature (8).

Conversely, other studies postulated that *SLC2A1* mutations alone might not be sufficient for the development of isolated migraine symptoms and they were not described in patients with HM as a unique clinical manifestation (9).

Our patient presented with mild psychomotor delay, as well as language development delay, which characterized the clinical picture for many years, together with ADHD-C. Epileptic history evolved from early-onset myoclonic-atonic seizures to atypical absence seizures. Thus, we could classify our patient as a mild or “non-classic” phenotype of GLUT1-DS, due to a *de novo* mutation in the *SLC2A1* gene. VPA in monotherapy was effective, with good clinical and EEG response.

During follow-up, interictal centro-temporal EEG abnormalities were interpreted as independent from the genetic mutations described and disappeared with age.

Later, she presented with non-epileptic paroxysmal events, characterized by involuntary distal athetotic movements, which are also a GLUT1-DS clinical manifestation, especially in mild phenotype (3).

In addition, the girl experienced other paroxysmal episodes, marked by transitory hemiplegia, often associated with headache. Such symptoms suggested the hypothesis of HM, leading to specific genetic testing, which revealed the pathogenetic mutation on the *CACNA1A* gene. Lamotrigine monotherapy was effective in preventing HM relapses over time.

Interestingly, our case presented with GLUT1-DS together with HM, due to *CACNA1A* mutation. To the best of our knowledge, the co-existence of such mutations in the same patient has not been described yet.

Our report highlights the importance of distinguishing between different PMDs classically experienced by patients with GLUT1-DS mild phenotype. Therefore, genetic testing, with the aim to achieve better etiological definition, is encouraged when transient hemiplegic events occur, to start specific treatment at an early stage.

## Data Availability Statement

The raw data supporting the conclusions of this article will be made available by the authors, without undue reservation, to any qualified researcher.

## Ethics Statement

Ethical review and approval was not required for the study on human participants in accordance with the local legislation and institutional requirements. The patients/participants provided their written informed consent to participate in this study.

## Author Contributions

CS: study design, execution, acquisition of data, drafting, writing, and revising. GM and SS: drafting, writing, and revising. MC, DS, and AC: drafting and revising. MF and GL: acquisition of data. SC: conceptual contribution, drafting, writing, and revising. All authors contributed to the article and approved the submitted version.

## Conflict of Interest

The authors declare that the research was conducted in the absence of any commercial or financial relationships that could be construed as a potential conflict of interest.
